# Thermo-/pH-dual responsive properties of hyperbranched polyethylenimine grafted by phenylalanine

**DOI:** 10.1007/s12272-013-0288-y

**Published:** 2013-11-22

**Authors:** Jie Chen, Jialiang Xia, Huayu Tian, Zhaohui Tang, Chaoliang He, Xuesi Chen

**Affiliations:** 1 Key Laboratory of Polymer Ecomaterials, Changchun Institute of Applied Chemistry, Chinese Academy of Sciences, Changchun, 130022 China; 2 College of Chemistry Science and Technology, Zhanjiang Normal University, Zhanjiang, 524048 China

**Keywords:** Amphiphilic polymers, Thermal- and pH-dual responsive, Phenylalanine, Polyethylenimine

## Abstract

Novel thermo- and pH-dual responsive amphiphilic copolymers were synthesized based on hyperbranched polyethylenimine (PEI) by grafting l-phenylalanine. The phenylalanine-modified PEI exhibited lower cytotoxicity than commercial PEI. These copolymers showed the phenomena of phase transitions in response to pH and temperature. The dilute copolymer solution at lower pH displayed the higher LCST. Furthermore, LCST increased with the increasing of phenylalanine grafting density. LCST of these copolymers were tunable from 7.2 to 59.6 °C by the degree of amidation and pH of solution. DLS and TEM experiments certified that the copolymer chains aggregated to form small size particles as increasing the temperature above LCST. For these reasons, the obtained smart copolymers were considered to be potential gene/drug carriers in biomedical field.

## Introduction

Polymers with stimuli-responsive property, such as fast and reversible changes in response to variations in temperature, pH, redox, light or electric fields, have attracted much interest in the past decade due to their medical and biological applications (Dobrynin and Rubinstein 2005; Kim and Oh 2005; Costa et al. 2009; Won et al. 2011; Liu et al. 2012; Yu et al. 2012). In particular, thermo- and pH-responsive polymers have been particularly attractive and intensively investigated for biomedical applications, including drug/gene delivery systems, injectable tissue engineering scaffolds, biosensors etc (Chaterji et al. 2007; Oda et al. 2010; Strozyk et al. 2012). It has been well known that some tissues and cellular compartments have different pH environments compared with normal physiological pH. Mean while, the temperature of specific sites in the body can be changed safely by hyperthermia, such as tumor tissues (Kojima et al. 2009; Alarcon et al. 2005; Schmaljohann 2006).

Thermo- and pH-responsive polymers exhibit the particularly attractive potential for biomedical applications because of the undergoing of a transition between water-soluble and water-insoluble states (Pang et al. 2011; Amin et al. 2012; Cha et al. 2012; Chen et al. 2013a). Among them, amphiphilic block copolymers containing amide segments have their natural property in thermo-sensitivity and the thermo-sensitivity can be adjusted by the number of amide bond, the ratios of hydrophilic and hydrophobic groups, and the length of hydrophilic and hydrophobic groups. Different response temperatures of the sensitive polymers have been synthesized by introducing a large number of amide bonds to hyperbranched poly(glycido). The LCSTs of polymers decrease with enhancing the hydrophobicity around the amide bond (Sakaguchi et al. 2008; Kojima et al. 2009). Typically, amino groups were utilized as pH-sensitive elements because of their ability of protonation/deprotonation. Liu and coworkers transformed all the end groups of hyperbranched PEI (HPEI) with propionyl chloride into isobutyramide groups, and obtained sensitive polymers both with thermo- and pH-sensitivity (Liu et al. 2007). However, the transition curves of light transmittance versus temperature were less sharp. Presumably, the entire primary amino of PEI was absolutely reacted, and the ability of protonation was limited due to the small amount of scanty tertiary amino groups. Using NHS/EDC·HCl condensation reaction, different hydrophobic amino acids were grafted onto poly(amidoamine) dendrimers, and the target polymers were provided with the ability of owing different range of thermo- and pH-sensitive (Haba et al. 2004, 2006; Tono et al. 2006).

Phenylalanine (Phenylalanine, abbreviated as Phe) is a natural amino acid with a hydrophobic phenyl group. Its carboxyl group can react with an amino group of another polymer and produce a temperature-sensitive amide bond. The amino group can provide pH sensitivity, and the phenyl group can provide hydrophobicity. Therefore, phenylalanine is a novel candidate to prepare thermo- and pH-dual sensitive polymers. However, very little attention has been paid to the application of phenylalanine in this regard (Tono et al. 2006; Casolaro et al. 2005). HPEI has exhibited many excellent performances due to the abundant levels of amino and their spatial structure (Park et al. 2008; Chen et al. 2009; Kim and Kim 2009; Tian et al. 2012). In 1995, PEI was successfully used for gene delivery (Boussif et al. 1995). Since then, a variety of structures based on PEI have been synthesized as candidates for the most efficient gene transfection reagents (Tian et al. 2007, 2013; Chen et al. 2013b). PEI can be easily prepared into biocompatible hydrogel (Lei et al. 2010). PEI can also absorb negatively charged materials in water due to its large number of positive charges. PEI can also be used in water treatment, paper industry, textile industry, oil drilling, and secondary recovery (Vicennati et al. 2008; Khaydarov et al. 2010).

In the present study, we synthesized a series of phenylalanine grafted PEI (abbreviated as PPhen, P means PEI, Phe means phenylalanine, and n means the molar feed ratio of phenylalanine to PEI) with thermo- and pH-dual sensitive properties. Subsequently, the cell viability of PPhen copolymers was evaluated compared with the commercial PEI. Furthermore, the factors that response to temperature and pH were investigated by controlling the relative proportions of amide segments.

## Materials and methods

### Materials

HPEI with a number average molecular weight of 10 kDa and weight-average molecular weight of 25 kDa (PEI25 K) was purchased from Aldrich. Boc-l-phenylalanine (Boc-Phe-OH) was purchased from Sigma and used as received. *N*-Hydroxysuccinimide (NHS) was purchased from Aesar Alfar (Ward Hill, MA, USA). Dimethylformamide (DMF) was treated with CaH_2_ and distilled before use. All other chemicals were purchased from Sigma-Aldrich (Munich, Germany). 3-(4,5-Dimethylthiazol-2-yl)-2,5-diphenyltetrazolium bromide (MTT) was purchased from Amresco (Solon, OH, USA). Dulbecco’s modified Eagle’s medium (DMEM) and fetal bovine serum (FBS) were purchased from Gibco (Grand Island, USA).

### Synthesis of PPhen copolymers

**Fig. 1:**
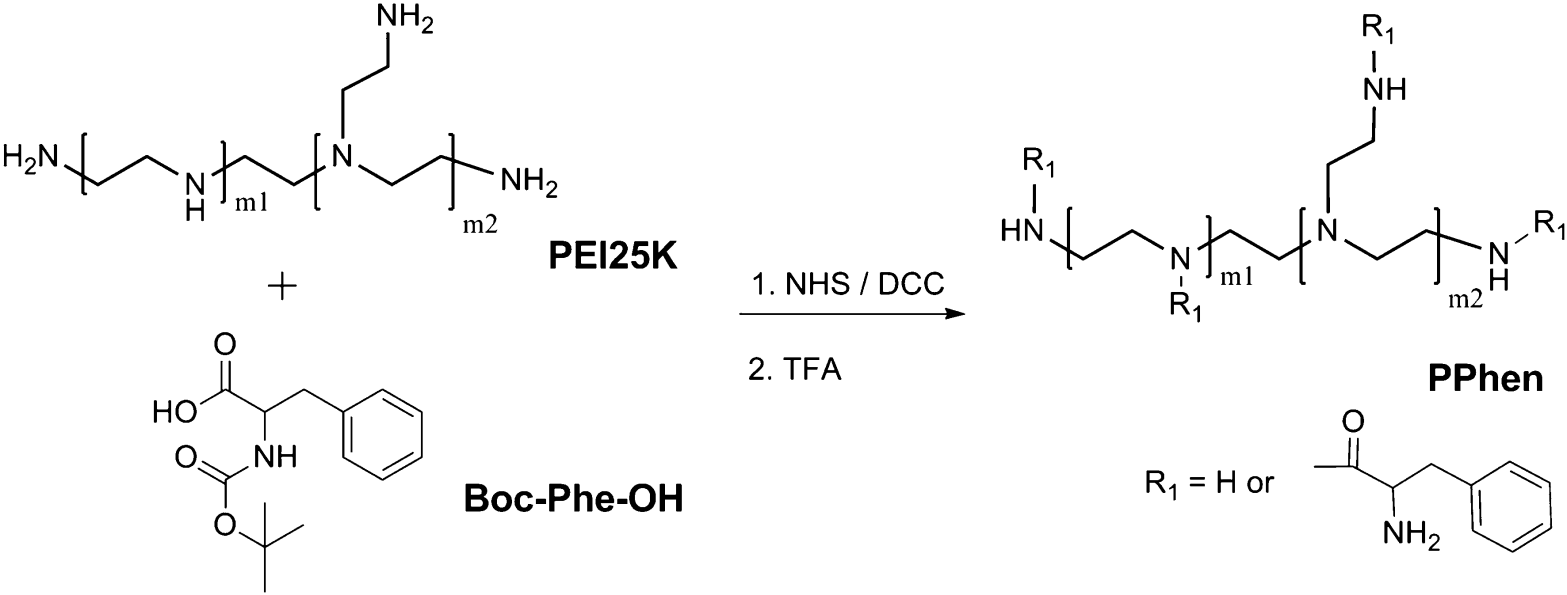
Synthesis of hyperbranched copolymers (PPhen)

A series of hyperbranched and amphiphilic PPhen copolymers were synthesized as shown in Fig. 1. Briefly, PEI25 K (2.0 g, 0.2 mmol), Boc-Phe-OH (0.2*n* mmol, *n* is the molar ratio of Boc-Phe-OH to PEI25 K), and NHS (0.4*n* mmol) were dissolved in dried DMF (30 mL). DCC (0.4*n* mmol) was added under the condition of ice bash after the powder was completely dissolved. The solution was stirred for 2 h at 0 °C, and then the temperature was increased to 25 °C for another 48 h. The solutions were precipitated twice with excess ethylether under vigorous stirring. Each PEI-(Phe-Boc)n copolymer was obtained after being dried under vacuum at room temperature.

The Butyloxycarbonyl groups in the PEI-(Phe-Boc)n were removed to obtain PPhen by TFA. Briefly, take the preparing of PPhe40 for instance, PEI-(Phe-Boc)40 (1.0 g) was dissolved in 5 mL of DMF at room temperature. Then 2 mL of TFA was added and stirred for 2 h. The solution was added dropwise into an excess of ethylether. The product was collected and dried under vacuum at room temperature overnight. The solid was dissolved in DMF and dialyzed (3,500 molecular weight cut off) against water. The final product (PPhe40) was collected by lyophilization.

Proton nuclear magnetic resonance (^1^H NMR) spectra were recorded in deuteroxide at room temperature by using a Bruker AV-400 NMR spectrometer (Bruker, Germany). Fourier-transform infrared (FT-IR) spectra of the PPhen copolymers were measured on a Vertex 70 FT-IR spectrometer (Bruker, Germany) using the KBr disk method.

### Cytotoxicity assay

Human epithelial carcinoma cells (HeLa cells) were cultured in DMEM medium, which was supplemented with 10 % (v/v) heat-inactivated FBS, 100 U/mL penicillin, and 100 μg/mL streptomycin in a 5 % CO_2_ incubator at 37 °C under 95 % humidity. HeLa cells were seeded in 96-well plates at a density of 1 × 10^4^ cells/well and incubated for 24 h prior to the treatment of polymer solutions with the concentration range from 0.015 to 1.0 mg/mL. After another 24 h, 20 μL of MTT solution (5 mg/mL in PBS) was added to each well. The MTT solution was carefully removed from each well after 4 h, and then 150 μL of DMSO was added to dissolve the MTT formazan crystals. The cell viability was analyzed by an ELISA microplate reader (Bio-Tek Instruments Inc., USA) according to the manufacturer’s protocol. The cell viability (%) was calculated according to the following equation:

$${\text{Cell viability}}\;(\%)=\left( {{\text{A}}_{\text{sample}} /{\text{A}}_{\text{control}}} \right) \times 100$$

where A_sample_ was the absorbance of the polymers treated cells and A_control_ was the absorbance of the untreated cells. Each experiment was performed as the average values of six runs and repeated a minimum of three times.

### Transmission electron microscopy (TEM) imaging of PPhen copolymers

TEM images were obtained using a TEM instrument (JEM-2010HR, Japan) in a 100 kV. To prepare the TEM samples, 0.1 μg/μL of the samples solution were pre-equilibrated at 25 and 40 °C. One drop of each sample was dropped onto a 300 mesh carbon-coated copper grid and dried at 25 and 40 °C, respectively.

### Measurement of phase transition

Phase transition measurements were carried out on a UV/vis spectrometer (Shimadzu UV-2401PC) equipped with a temperature controller (Shimadzu S-1700). Briefly, the turbidity of solutions of PPhen was measured at 500 nm in 50 mM phosphate containing 150 mM NaCl buffer, pH 6.8–12.2. The heating rate of the sample cell was maintained at 2 °C/min over the temperature range of 5–75 °C. The concentration of the polymers was 2 mg/mL. The LCST was defined as the temperature corresponding to the initial break points in the resulting transmittance versus temperature curves. (Liu et al. 2007; Zhang et al. 2011)

## Results and discussion

### Synthesis and characterization of the PPhen copolymers

**Fig. 2:**
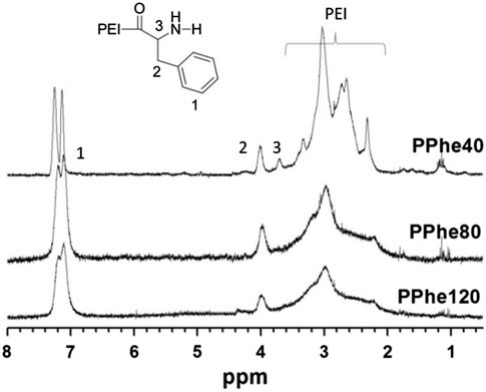
^1^H NMR spectra of the PPhen copolymers in D_2_O

The synthetic route for the preparation of PPhen copolymer was outlined in Fig. 1. The feed molar ratios of Boc-Phe-OH to PEI25 K were 40, 80, and 120, respectively. All the PPhen copolymers were well water soluble. The ^1^H NMR spectra of the PPhen copolymers are presented in Fig. 2. The number of grafted Phe segments in the copolymers (n_(Phe in PPhe)_) was estimated by ^1^H NMR spectroscopy (Table 1), and n_(Phe in PPhe)_ was calculated according to the following formula:

$${\text{n}}_{{({\text{Phe\,in\,PPhen}})}} = \frac{10000}{43} \times \frac{{4 \times \varvec{ I}_{\delta = 7.18} }}{{5\varvec{ } \times \varvec{ I}_{\delta = 2.3\sim 3.4} }}$$

**Table 1:** Characterization of PPhen copolymers

Sample	Molar ratio of Phe/PEI		Phe content in PPhen (%)	
	Theoretical^a^	Calculative	Theoretical^a^	Calculative
PPhe120	120:1	88:1	63.8	56.4
PPhe80	80:1	77:1	54.0	53.1
PPhe40	40:1	38:1	37.0	35.8

^**a**^The theoretical value was calculated based on PEI25 K with a number average molecular weight of 10 kDa

**Fig. 3:**
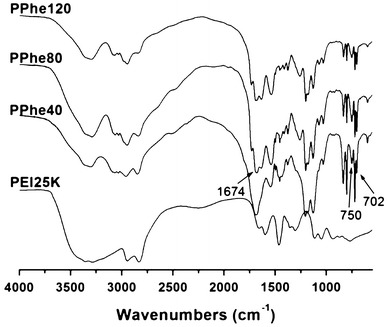
The FT-IR spectra of PEI25 K and PPhen copolymers

The FT-IR spectra of PEI25 K and PPhen copolymers are shown in Fig. 3. In the spectra, the absorption peak of amide groups at 1,674 cm^−1^ (HN–C=O) and the peaks at 702 and 750 cm^−1^ of monosubstituted benzene in the fingerprint indicated that the thermo- and pH-sensitive copolymers were successfully prepared.

HPEI contains primary, secondary, and tertiary amines, and the molar ratios of them for PEI25 K were 31:39:30 according to their respective inverted gate ^13^C NMR spectra. (Liu et al. 2007) In other words, a PEI25 K molecule contains the numbers of primary, secondary, and tertiary amines were 72, 90, and 70, respectively. Primary amines of HPEI are much more reactive than the secondary amines. When the feed molar ratios of Boc-Phe-OH to PEI25 K were 40 and 80, about 56 and 89 % of the primary amines in HPEI were reacted to form amide bands, respectively. When the feed molar ratio was increased to 120, all the primary amines were reacted completely and some secondary amines also participated in the reaction.

### Cytotoxicity of PPhen copolymers

**Fig. 4:**
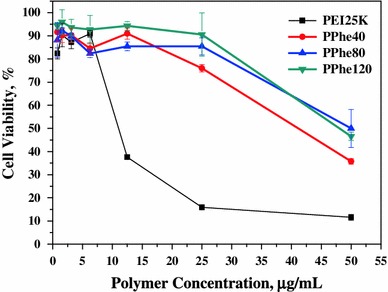
Viability of HeLa cells exposed to various polymer concentrations for PEI25 K and PPhen

The cytotoxicity of PEI depends on the density of positive charges on its surface. The PPhen copolymers exhibited relatively low cytotoxicity for potential medical applications, as compared with PEI25 K (Fig. 4). The improved cytocompatibility of PPhen copolymers may be due to the introduction of biocompatible and hydrophobic Phe moieties, which shield the high positive charge density on the surface of PEI.

### Thermo- and pH-responsive properties of PPhen copolymers

**Fig. 5:**
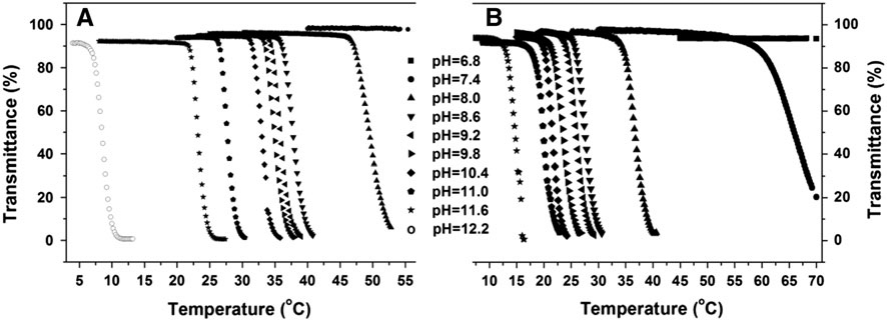
Effect of temperature on the phase transition behavior of PPhe80 (**a**) and PPhe120 (**b**) at different pH values

The phase transition behaviors of the copolymers were characterized by turbidimetry measurement on a UV/Vis spectrometer with temperature-controlled source at 500 nm. The LCST was defined as the temperature corresponding to 90 % transmittance of aqueous solution during the heating process. Figure 5 showed at different pH condition, the transmittance change of PPhe80 and PPhe120 in aqueous solutions with a constant heating rate of 2 °C min^−1^. As shown, the transmittance decreased dramatically in response to the temperature change around the LCST, indicating the highly sensitive phase separation of PPhe80 and PPhe120 copolymers.

With introducing of amide bond, the temperature-sensitive property could be bestowed to PEI25 K. Hydrogen bonds, formed by amide bonds, amino groups and H_2_O could provide good hydrophilicity of copolymers, and the hydrophilicity would be weakened by temperature rising. The phenyl groups of Phe could provide fixed hydrophobic interaction. Furthermore, the lower pH there was, the more amino groups protonated, which provide very strong hydrophilic property. Thus, PPhe80 copolymer had a dual responsive property with the change of temperature and pH. As Fig. 5 and Table 2 showed, PPhe80 showed the lower pH, the higher LCST. PPhe80 exhibited rapid temperature response between the pH values of 8.0–12.2. And with the pH value increased, LCST reduced from 47.4 to 7.2 °C. When the pH value exceeded 11, PPhe80 copolymer was not soluble in water at room temperature.

**Table 2:** The LCST (°C) of PPhen copolymers at different pH values

Polymers	pH									
	6.8	7.4	8.0	8.6	9.2	9.8	10.4	11.0	11.6	12.2
PPhe120	–	59.6	34.8	25.8	24.2	21.8	20.2	18.8	13.2	–
PPhe80	–	–	47.4	36.4	34.6	33.8	31.8	26.8	22.2	7.2
PPhe40	–	–	–	–	–	–	–	–	–	–

(–) indicates that LCSTs could not be detected at their corresponding pH values, in the tested temperature range

The influence of the relative proportions of Phe groups and PEI25 K were also investigated. Different phenylalanine grafting density of PPhe40, PPhe80 and PPhe120 were prepared by adjusting the feed ratio of Boc-Phe-OH and PEI25 K. The LCSTs of PPhe40, PPhe80 and PPhe120 at different pH values were summarized in Table 2. Figure 5b showed that, with similar properties of PPhe80, the PPhe120 solution exhibited rapid temperature response. LCST of the PPhe120 solution reduced from 59.6 to 13.2 °C with pH values increased from 7.4 to 11.6. Compared the results of Table 2, LCST of PPhe120 was lower than that of PPhe80 at the same pH value. PPhe120 has more benzene rings and amide bonds than PPhe80, so that the lower temperature could make the hydrophobic effect played a dominant role. For PPhe40 copolymer, there was only a few of benzene rings and amide bonds, the protonation ability and hydration were overwhelmingly dominant, so it did not exist LCST transition.

**Fig. 6:**
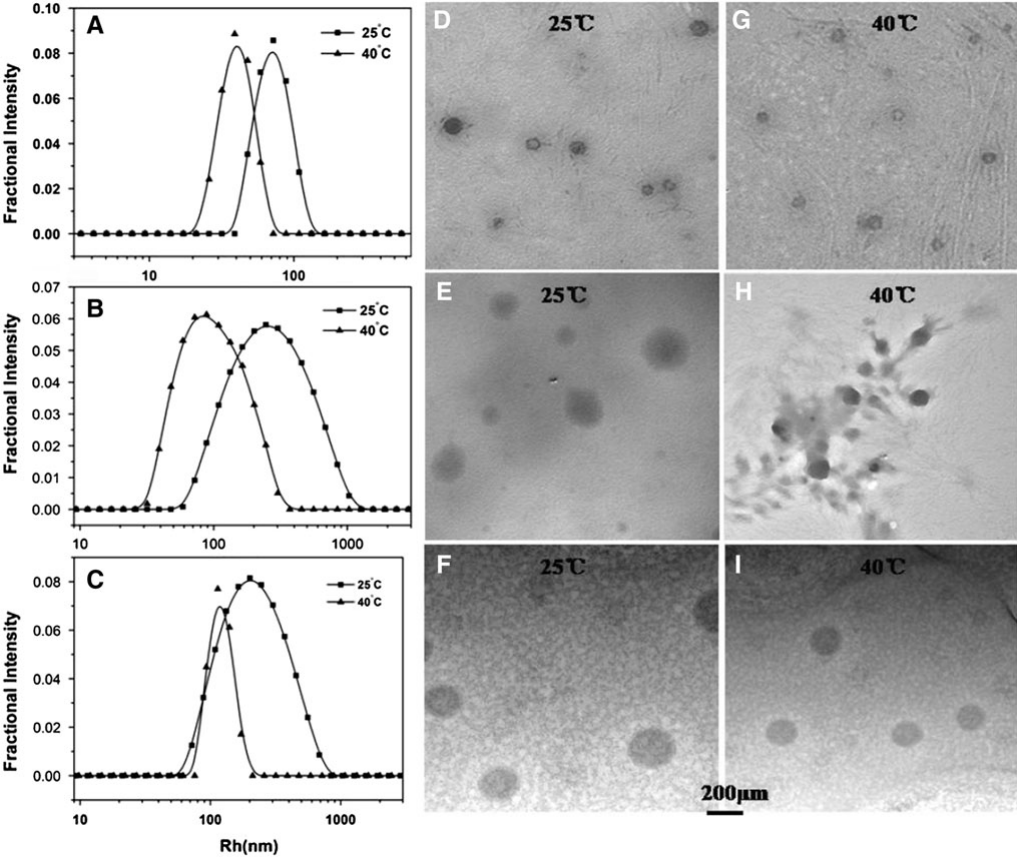
The representative size distribution curves of PPhe40 (**a**), PPhe80 (**b**), and PPhe120 (**c**) at 0.5 mg/mL at pH9.8, pH9.8, and pH8.0, respectively. The rectangular and triangular symbols denote the data collected at 25 and 40 °C. TEM images of PPhe40 (**d** and **g**), PPhe80 (**e** and **h**), and PPhe120 (**f** and **i**) at pH9.8, pH9.8, and pH8.0, respectively, with a concentration of 0.1 mg/mL at different temperatures

Figure 6a–c showed the size distributions of PPhen copolymers at 0.5 mg/mL with increasing temperature at different pHs by DLS assay. LCST of PPhe80 was 33.8 °C at pH9.8. When the temperature was 25 °C, the average particle size of PPhe80 was 246 nm. (Fig. 6b) Under this condition, the PPhe80 copolymer was in a relatively loose aggregation state because of the dominant position of hydrophilic interaction. However, when the temperature was 40 °C, the average particle size of PPhe80 was 88.5 nm. The hydrophobic interaction was in a good condition at this time. The aggregation state of PPhe80 was further compressed in the hydrophobic effect caused by benzene rings and amide bonds. LCST of PPhe120 was 34.8 °C at pH8.0. Figure 6c showed that the average particle size of PPhe120 was 201 nm, when the temperature was 25 °C, while the particle size shrank to 114 nm at 40 °C. That was similar to PPhe80, but PPhe120 copolymer was more hydrophobic, and the distribution of particle size became narrower above LCST. For the copolymer of PPhe40, (Fig. 6a) with the increase of temperature, the hydrogen was destroyed gradually, and the particle size was compressed from 72.2 to 39 nm because of the hydrophobic interaction of benzene rings and amide bonds. Meanwhile, there had little change in the particle size distribution width before and after heating, probably due to the weakly hydrophobic interaction of PPhe40. The particle size of PPhe40 could shift with temperature variation, which showed that PPhe40 copolymer also had certain sensitivity to temperature, but the sensitivity was much weaker than that of PPhe80 and PPhe120. Figure 6(d–i) showed the TEM images of PPhen copolymers obtained by the self-assembly in aqueous solution with increasing temperature at different pHs values. There were consistent results with DLS assay, the particle sizes of aggregates at 25 °C were larger than those at 40 °C, which could also indicate that the PPhen copolymers had thermo-responsive properties.

## Conclusion

In this study, thermo- and pH dual responsive PPhen copolymers were successfully synthesized by hydrophobic phenylalanine grafting of HPEI. The improved cytocompatibility of PPhen copolymers was due to the introduction of biocompatible Phe moieties, which shielded the high positive charge density on the surface of PEI. The obtained copolymers showed LCST in aqueous solution. LCSTs of these copolymers could be altered from 7.2 to 59.6 °C by varying the degree of amidation by phenylalanine groups and pH of solution. The obtained thermo- and pH dual responsive copolymers might be more suitable for being used as ‘smart’ polymers in biomedical and biotechnological fields.
